# Vectors and Spatial Patterns of* Angiostrongylus cantonensis* in Selected Rice-Farming Villages of Muñoz, Nueva Ecija, Philippines

**DOI:** 10.1155/2016/3085639

**Published:** 2016-05-24

**Authors:** Ma. Angelica A. Tujan, Ian Kendrich C. Fontanilla, Vachel Gay V. Paller

**Affiliations:** ^1^Animal Biology Division, Institute of Biological Sciences, College of Arts and Sciences, University of the Philippines Los Baños, 4031 Laguna, Philippines; ^2^DNA Barcoding Laboratory, Institute of Biology, College of Science, University of the Philippines Diliman, 1001 Quezon City, Philippines

## Abstract

In the Philippines, rats and snails abound in agricultural areas as pests and source of food for some of the local people which poses risks of parasite transmission to humans such as* Angiostrongylus cantonensis*. This study was conducted to determine the extent of* A. cantonensis* infection among rats and snails collected from rice-farming villages of Muñoz, Nueva Ecija. A total of 209 rats, 781 freshwater snails, and 120 terrestrial snails were collected for the study. Heart and lungs of rats and snail tissues were examined and subjected to artificial digestion for parasite collection. Adult worms from rats were identified using SSU rDNA gene. Seven nematode sequences obtained matched* A. cantonensis*. Results revealed that 31% of the rats examined were positive with* A. cantonensis*.* Rattus norvegicus* and* R. tanezumi* showed prevalence of 46% and 29%, respectively. Furthermore, only* Pomacea canaliculata* (2%) and* Melanoides maculata* (1%) were found to be positive for* A. cantonensis* among the snails collected. Analysis of host distribution showed overlapping habitats of rats and snails as well as residential and agricultural areas indicating risks to public health. This study presents a possible route of human infection for* A. cantonensis* through handling and consumption of* P. canaliculata* and* M. maculata* or crops contaminated by these snails.

## 1. Introduction

*Angiostrongylus cantonensis* or the rat lungworm is a zoonotic helminth responsible for the disease called angiostrongylosis. Its life cycle involves rodents as definitive hosts and mollusks as intermediate hosts. It can also infect other animals, for example, shrimps and frogs, without further development and still be infective when ingested. Humans, however, are dead-end hosts for* A. cantonensis* and can be infected through ingestion of infected mollusks, things contaminated by infected mollusks, for example, soil and vegetables [1, 2], and ingestion of paratenic hosts. As a result,* A. cantonensis* is the major cause of eosinophilic meningitis in humans particularly in Indo-Pacific regions where it is endemic. The animal-human environmental interface of* A. cantonensis* is difficult to assess and one of the reasons is that its hosts are easily affected by changes in the environment [3]. Changes in ecology and environment may also result in changes in the epidemiology of this parasite. Thus, it is important to assess the possible transmission route of the parasite due to its risks to both veterinary and public health.

Nueva Ecija is the rice granary of the Philippines and one of its towns, Muñoz, was observed to have* A. cantonensis* [4, 5]. The intensity and molecular biology of the observed parasites were not determined in previous studies. However, it is important to present stronger evidence regarding the presence of* A. cantonensis* as it could be mistaken for other species of* Angiostrongylus*. Furthermore, the intermediate host remains unknown in the region. This is an important key in assessing the infection of* A. cantonensis* particularly in humans because these hosts harbor the infective stage of the parasite. Thus, this study was conducted to determine the extent of infection of* A. cantonensis* among rats and snails collected from Muñoz.

## 2. Materials and Methods

### 2.1. Study Site

Muñoz is located in Nueva Ecija, Philippines, comprising of 37 villages with a total population of 85,461. It is globally positioned at 15.71°N latitude and 120.90° longitude E and has a total land area of 16,305 hectares which is mainly for agricultural utilization (9,819 hectares) followed by residential zone (2,847 ha) [6]. Five rice-farming villages were randomly selected for the study during June 2014-October 2015.

### 2.2. Harvest of* Angiostrongylus cantonensis* in Rats

Single live capture traps were used for the collection of rats. Rats were euthanized and dissected for the presence of* A. cantonensis*. The heart and lungs of the rat were examined for adult worms. Collected worms were preserved in 100% ethanol. Adult* A. cantonensis* were identified with the female having a barber pole appearance and male having a copulatory bursa [7]. Additionally, the organs were artificially digested with pepsin-HCl solution in a hot plate with magnetic stirrer at 37°C ± 2°C for one hour. It was filtered and placed in a petri dish for microscopic examination of larvae.

### 2.3. DNA Extraction, PCR Amplification, and Sequencing of Adult Worms Recovered from Rats

The identification of obtained worms was performed using the standard molecular barcode of soil nematodes, the 5′ end of the small subunit ribosomal RNA gene (SSU rDNA) [8–10]. Total genomic DNA was extracted from adult worms using PureLink® Genomic DNA (Life Technologies) kit. The SSU rDNA gene was amplified through polymerase chain reaction (PCR) using the following primers: SSU_F_07 (sense) 5′-AAAGATTAAGCCATGCATG-3′ and SSU_R_09 (anti-sense) 5′-AGCTGGAATTACCGCGGCTG-3′ [10]. A total of 50 *μ*L of PCR mix was prepared consisting of 5 *μ*L PCR buffer with 1.5 mM MgCl_2_, 1.0 *μ*L 10 mM dNTP, 2.5 *μ*L 10 *μ*M of each primer, 10 *μ*L Q buffer (Qiagen, Netherlands), 0.25 *μ*L 1.25 T Taq (Roche*™*, USA), and 4 *μ*L DNA sample. The amplification was performed using Labnet MultiGene*™* thermocycler with PCR conditions of 94°C for three minutes, 43 cycles of 94°C for 30 seconds, 45°C for 30 seconds, and 65°C for one minute, the final extension at 72°C for five minutes. PCR products were visualized in 1% agarose gel with ethidium bromide under ultraviolet illumination. Qiagen*™* Gel Extraction Kit (USA) was used to extract the PCR products from the gel. The purified PCR products were sent to 1st Base, Malaysia, for sequencing of the antisense strands. DNA sequences were assembled using STADEN package version 1.5.3 [11] and aligned using BioEdit Sequence Alignment Editor 7.0.9.0 [12]. The species with the closest SSU rDNA sequence from GenBank for each nematode sequence was determined using the nucleotide Basic Local Alignment Search Tool (BLAST, http://blast.ncbi.nlm.nih.gov/Blast.cgi/, [13]).

### 2.4. Collection of Potential Intermediate Snail Hosts

Freshwater snails were handpicked from rivers, rice fields, irrigations, and around houses while terrestrial snails were handpicked along walls and trees. All collected snails were placed in labeled containers and transferred to the laboratory for identification and parasite examination. Each snail was chopped into small pieces and placed in a petri dish containing Ash's digestive fluid [14]. It was left overnight at 37°C and live larvae were observed under a microscope.* A. cantonensis *third stage larvae (L3) were identified with their two chitinous rods at the anterior end and a slightly curved and pointed tail [7].

### 2.5. Survey on Knowledge and Practices of Locals on Parasites from Rats and Snails

One hundred respondents in the study site were interviewed and a questionnaire was given out to each respondent. The respondents include farmers and housewives which are people that have high chances of getting* A. cantonensis.*

### 2.6. Data Analyses

Prevalence and mean intensity were computed for both rats and snails. Index of discrepancy was used to determine the distribution pattern of* A. cantonensis* in both rat species [15]. Furthermore, chi-square test of independence and Fisher's exact test were used to compare the prevalence between rat species whereas Mann Whitney *U* test was used to compare intensities between rat species. All statistical analyses were performed at 95% confidence level using several statistical analysis software programs including Quantitative Parasitology version 3.0 and Predictive Analysis Software version 18.0 both for Windows.

## 3. Results

A total of 209 rats, 781 freshwater snails, and 120 terrestrial snails were collected and examined for* A. cantonensis* infection. The rats were identified as* R. norvegicus* (*n* = 24) and* R. tanezumi* (*n* = 185). The freshwater snails were grouped according to their species:* Indoplanorbis exustus* (*n* = 14),* Jagora asperata* (*n* = 7),* Melanoides maculata* (*n* = 89),* Pomacea canaliculata* (*n* = 200),* Radix quadrasi* (*n* = 138),* Tarebia granifera* (*n* = 108),* Vivipara angularis* (*n* = 41), and* Vivipara carinata* (*n* = 184). Moreover, all terrestrial snails collected were identified as* Achatina fulica* (*n* = 120).

### 3.1. SSU rDNA Gene Sequences Detected in Adult Worms from Rats

Seven representative adult female worms were successfully subjected to DNA extraction, PCR amplification, and sequencing. Floyd et al. [8] proposed that two sequences belong to the same species when they are 99.5–100% identical for the 450 bp of the 5′ end of the SSU rDNA gene. Thus,* A. cantonensis* was identified with certainty to species level based on GenBank BLAST results (Table 1).

### 3.2. *Angiostrongylus cantonensis* Infection in Rats

A total of 64 rats (31%) were found to be infected with* A. cantonensis* (Table 2). The rats belonging to* R. norvegicus* showed 46% (11/24) prevalence for* A. cantonensis* infection while* R. tanezumi* showed 29% (53/185) prevalence. Statistical analysis revealed no significant differences between the rat species (*χ* ^2^ = 0.086, *P* > 0.05). Furthermore, intensity of* A. cantonensis* for* R. tanezumi* (62 parasite/rat) is higher than* R. norvegicus* (43 parasite/rat) and also showed no significant differences (*U* = 286.5; *P* > 0.05).

### 3.3. *Angiostrongylus cantonensis* from Snails

A total of 84 snails (9%) were found infected with nematode larvae (Table 3). However, only* M. maculata* (1/89) and* P. canaliculata* (3/200) harbored the* A. cantonensis* L3. These infected snails were collected from rice fields and irrigation.

### 3.4. Distribution of Rats and Snails in Muñoz

**Figure 1:**
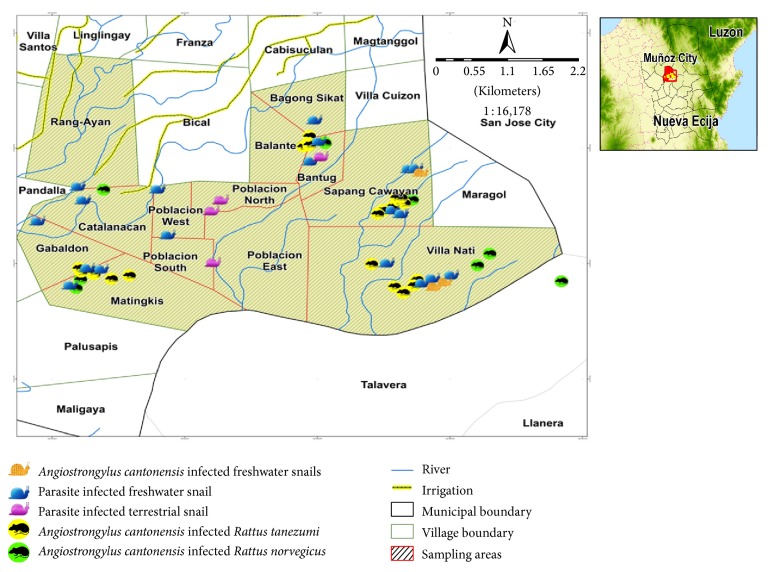
Map of Muñoz, Nueva Ecija, showing the distribution of* Angiostrongylus cantonensis* infected rats and snails.

The distribution map revealed that the sampling points for infected rats and snails overlap implying potential hosts occurring in the same area (Figure 1). The map also showed that the selected villages have rats and snails harboring* A. cantonensis* and other parasites. Two villages, namely, Sapang Cawayan and Villa Nati have the known definitive and intermediate hosts of* A. cantonensis*. However, there are also villages which have* A. cantonensis* infected rats but no* A. cantonensis* infected snails.

### 3.5. Knowledge and Practices of Locals on Parasites from Rats and Snails

One hundred respondents from Muñoz consisting of adult males (*n* = 72) and females (*n* = 28) were interviewed for the study (Table 4). According to the survey, most of the respondents eat rat. The local people believe that it cures skin diseases. Apparently, most of the locals have eaten rat meat once or twice especially when they were still young. Some of them also made their children eat rat meat primarily because of its presumed medicinal property. Meanwhile, most of the locals usually eat* R. quadrasi*,* M. maculata*, and* Vivipara* species and a few of them eat* P. canaliculata*. In addition, locals are not aware that ingestion of snails with parasites can cause diseases in humans.

## 4. Discussion

Transmission of* Angiostrongylus cantonensis* is very complex and difficult to assess. It involves parasitizing both warm- and cold-blooded animals which do not necessarily share the same spatial distribution and ecological requirements [16]. Furthermore, its larval stages are easily affected by different environmental factors such as temperature, oxygen, and pH as well as other factors such as host range and spatial and temporal variations. However, there is a need to further investigate its transmission in order to understand how to control and prevent its associated disease.

The importance of fast and accurate diagnostic tools for diseases caused by nematodes lies on the knowledge of their prevalence and geographical distribution [17] which is specifically true for* A. cantonensis* having nearly all mollusks as its intermediate host. Moreover, there are two other rat lungworms that have similar life cycle with* A. cantonensis*,* A. malaysiensis,* and* A. mackerrasae*. Most diagnoses of these nematodes are based on morphological characteristics; however, specific identification of these nematodes is unfeasible because of the similar descriptions on the size and body shapes among its species [17]. Hence, misidentification and misdiagnoses of these nematodes may result in the underestimation of their infections. That is why most works on nematodes have chosen rDNA regions because they are useful genetic markers for studies on diagnosis, systematics, and molecular evolution [17]. Thus, utilization of genetic markers is widely used in differentiating closely related species like in the case of* Angiostrongylus*.

In this study, the sampling sites were mainly comprised of agricultural areas where* R. tanezumi* is abundant. Moreover, these sites are located in rice fields and water reservoirs which are perfect habitat for snails.* R. tanezumi* primarily consume insects, snails, slugs, and other invertebrates found in their habitats and they specifically survive on frogs and snails during non-rice periods [5]. This may have resulted in the increased contact of* R. tanezumi* with possible intermediate hosts of* A. cantonensis* leading to the transmission of the parasite. On the other hand,* R. norvegicus* is usually located in cities and towns and is less commonly found in cultivated areas [18]. Nevertheless, other factors may have also contributed to the prevalence and intensity of* A. cantonensis* in both rat species such as the exposure of these rats to the intermediate hosts, behavior, and feeding habits of rats, as well as their genetic traits.

The low prevalence of* A. cantonensis* in freshwater snails in the study may has resulted from a number of factors such as the seasonal infection of rats with* A. cantonensis*. Based on a study by Antolin et al. [4], female* R. tanezumi* in PhilRice farms from Nueva Ecija were found to be infected with* A. cantonensis* during June to September. This could mean that the transmission of the parasite between its hosts may have happened during these months. In connection to this, snails for the study were collected during other months of the year. Furthermore, ecological characteristics of* P. canaliculata* and* M. maculata* such as their benthic life cycle may also be accounted for the low prevalence of* A. cantonensis* infection. According to Lv et al. [16], only a few species of freshwater snails naturally transmit* A. cantonensis* because rat feces containing its L1 are diluted in freshwater bodies. Previous studies suggest that* A. cantonensis* infection in terrestrial snails and slugs are higher than freshwater snails [19–21]. In the present study, no* A. cantonensis* L3 was observed in* A. fulica*. The reason is unclear; however, possibilities exist such as effects of seasonal variations and habitat of hosts on the transmission of parasite. Based on observations,* A. fulica* becomes active when it starts to rain. In the study of Salibay and Luyon [22], fewer rats were caught in rainy days. This indicates that there may have been less contact between rats and* A. fulica*. Studies in other countries have shown that* A. fulica* are naturally infected with the parasite [8, 17, 19, 23–25]. In the Philippines, few reports regarding* A. cantonensis* infection in snails can be found.* A. fulica* and* Laevicaulis altae* from Metro Manila were revealed to be infected with* A. cantonensis* [9, 10, 26] but other than the snail, no other reports have been made. This study, however, is the first record of* A. cantonensis* infected* P. canaliculata* and* M. maculata* in the Philippines.

Maps may be used to predict the probability of animals occurring in an area [27–29] and in this case the rats and snails. Rodents exhibit territorial behavior [30]; however, both rat species have overlapping sampling points in the study. This may be due to residential areas being adjacent to agricultural lands.* R. norvegicus* is a major urban pest worldwide but it was also reported as a field pest in some parts of the Philippines. They are also often found close to water sources like rivers and irrigations [18]. Furthermore, Salibay and Luyon [22] revealed in their study that* R. norvegicus* were mostly observed in areas where* R. tanezumi* were also caught. The unusual change in habitat of* R. norvegicus* from residential to agricultural is probably due to habitat alterations. Moreover, food consumption of rats can also be accounted for the captured rat distribution. When food is not available in their habitat, rats would either resort to cannibalism [5] or look for other sources of food. Meanwhile, snails were collected in rice fields, waterways, and near houses which can be easily accessed by rats. The locations of* A. cantonensis* infected snails coincide with those of* A. cantonensis *infected rats. Hence, transmission of parasites between rats and snails has been occurring in the study sites.

Eosinophilic meningitis is caused by several helminth and nonhelminth parasites [31] but the most common cause is* A. cantonensis* [32] which is linked to the introduction, farming, and consumption of some snail species. Meningitis was recorded as one of the leading causes of child mortality in the Philippines from 2001 to 2006 and 2008 to 2010 [33]. The analysis of cerebrospinal fluid (CSF) as well as blood culture aids in their proper classification of CNS infections; however, it may not always be followed because of the limitations on the part of the patient. Thus, the final diagnosis is dependent on the assessment and opinion of the attending physician [34]. Even though the cases of meningitis were not solely based on* A. cantonensis* infection alone, the prevalence of the parasite is of concern and even more if diagnosis of the parasite in humans is neglected most of the times. In conclusion, human cases of angiostrongylosis have not been recorded in Muñoz but this could be due to the misdiagnoses and lack of readily available diagnostic tools. The presence of the parasite in potential hosts could not eliminate the possibility of its transmission to humans. Hence, public education regarding zoonotic parasites should be implemented. Proper handling of its intermediate hosts and crops that may be contaminated by its hosts should be practiced. Moreover,* P. canaliculata* and* M. maculata* should be further examined since it is in these populations that larvae of* A. cantonensis* were observed. Other regions in the Philippines should also be evaluated particularly in those areas where rice planting is the main source of livelihood.

## Figures and Tables

**Table 1:** Adult worm sequences obtained in the study and their closest match in GenBank.

Sequence	Closest match	Identity
RN (4BDYKU3S01R)	*Angiostrongylus cantonensis*	100%
RT1 (4BEG550U016)	*Angiostrongylus cantonensis*	99.8%
RT2 (4BER379J016)	*Angiostrongylus cantonensis*	100%
RT3 (4BEW135B016)	*Angiostrongylus cantonensis*	100%
RT4 (4BEYNXB6016)	*Angiostrongylus cantonensis*	99.8%
RT5 (4FJJJW6R013)	*Angiostrongylus cantonensis*	99.8%
RT6 (4FJNEHJH013)	*Angiostrongylus cantonensis*	99.8%

**Table 2:** Rats examined for *Angiostrongylus cantonensis* infection in Muñoz, Nueva Ecija.

Rats	Sites					Total	Prevalence (%)
	Bantug	Catalanacan	Matingkis	Sapang Cawayan	Villa Nati		
*Rattus norvegicus *	8 (4)	3 (1)	5 (2)	4 (1)	4 (3)	**24 (11)**	**46**
*Rattus tanezumi *	34 (13)	31 (1)	28 (11)	54 (14)	38 (14)	**185 (53)**	**29**
Total	**42 (17)**	**34 (2)**	**33 (13)**	**58 (15)**	**42 (17)**	**209 (64)**	**31**

**(a):** 

Snails	Sites					Total	*Angiostrongylus cantonensis*	Infected with other nematodes
	Bantug	Catalanacan	Matingkis	Sapang Cawayan	Villa Nati			
*Freshwater*								
*Indoplanorbis exustus *	1	1	10	0	2	14	−	−
*Jagora asperata*	2	1	0	4	0	7	−	−
*Melanoides maculata *	20	18	12	21 (1)	18	89	+	−
*Pomacea canaliculata*	25	37	37	76 (1)	25 (2)	200	+	+
*Radix quadrasi*	22	24	39	20	33	138	−	+
*Tarebia granifera*	13	7	0	88	0	108	−	+
*Vivipara angularis*	1	0	25	7	8	41	−	+
*Vivipara carinata*	31	29	15	66	43	184	−	+
Total	**115**	**117**	**138**	**282**	**129**	**781**	** **	** **

**(b):** 

Snails	Sites				Total	*Angiostrongylus cantonensis*	Infected with other nematodes
	Bagong Sicat	Poblacion East	Poblacion North	Poblacion South			

*Terrestrial*							
*Achatina fulica*	30	30	30	30	120	−	+

**Table 4:** Response of the local people in Muñoz, Nueva Ecija regarding awareness on parasitic diseases from rats and snails.

Question	Response (%)
Eat rats	
Yes	60
No	40
Difference of rat species	
Yes	98
No	2
Eat snails	
Yes	84
No	16
Snail species as food	
Birabid (*R. quadrasi*)	41
Susong pilipit/palipit (*M. maculata*)	71
Susong papa (*Vivipara* sp.)	69
Golden apple snail (*P. canaliculata*)	15
Source of snail	
River	52
Irrigation	53
Rice field	40
Fish Pond	20
Small creek	16
Other animals from the field as source of food	
Frog	53
Igat (eel)	35
Tulya (shellfish)	77
Fish	11
Talangka (crab)	20
Awareness of disease associated with eating snails	
Yes	5
No	95
